# Role of Organic Anions and Phosphatase Enzymes in Phosphorus Acquisition in the Rhizospheres of Legumes and Grasses Grown in a Low Phosphorus Pasture Soil

**DOI:** 10.3390/plants9091185

**Published:** 2020-09-11

**Authors:** Driss Touhami, Richard W. McDowell, Leo M. Condron

**Affiliations:** 1 Faculty of Agriculture and Life Sciences, P.O. Box 85084, Lincoln University, Lincoln 7647, Christchurch, New Zealand; Richard.mcdowell@agresearch.co.nz (R.W.M.); Leo.Condron@lincoln.ac.nz (L.M.C.); 2 AgroBioSciences Program, Mohammed VI Polytechnic University (UM6P), Hay Moulay Rachid, Ben Guerir 43150, Morocco; 3 AgResearch Limited, Lincoln Research Centre, Private Bag 4749, Christchurch, New Zealand

**Keywords:** phosphorus acquisition, organic anions, phosphatase enzymes, rhizosphere P fractions, legumes, grasses

## Abstract

Rhizosphere processes play a critical role in phosphorus (P) acquisition by plants and microbes, especially under P-limited conditions. Here, we investigated the impacts of nutrient addition and plant species on plant growth, rhizosphere processes, and soil P dynamics. In a glasshouse experiment, blue lupin (*Lupinus angustifolius*), white clover (*Trifolium repens* L.), perennial ryegrass (*Lolium perenne* L.), and wheat (*Triticum aestivum* L.) were grown in a low-P pasture soil for 8 weeks with and without the single and combined addition of P (33 mg kg^−1^) and nitrogen (200 mg kg^−1^). Phosphorus addition increased plant biomass and total P content across plant species, as well as microbial biomass P in white clover and ryegrass. Alkaline phosphatase activity was higher for blue lupin. Legumes showed higher concentrations of organic anions compared to grasses. After P addition, the concentrations of organic anions increased by 11-,10-, 5-, and 2-fold in the rhizospheres of blue lupin, white clover, wheat, and ryegrass, respectively. Despite the differences in their chemical availability (as assessed by P fractionation), moderately labile inorganic P and stable organic P were the most depleted fractions by the four plant species. Inorganic P fractions were depleted similarly between the four plant species, while blue lupin exhibited a strong depletion of stable organic P. Our findings suggest that organic anions were not related to the acquisition of inorganic P for legumes and grasses. At the same time, alkaline phosphatase activity was associated with the mobilization of stable organic P for blue lupin.

## 1. Introduction

From gene duplication to root system development and photosynthesis, phosphorus (P) is involved in a plethora of biological and biochemical mechanisms that are critical to crop production [1,2]. The total amount of P in soils may exceed plant requirements; however, only a small amount is available for plant uptake [3]. Soil P is present in inorganic and organic forms [4]. Inorganic P is subject to adsorption and precipitation with iron (Fe), aluminum (Al), and calcium (Ca) cations, as well as with positively charged soil particles [5,6], whereas organic P represents up to 65% of total P and requires a mineralization step to become available for plant uptake [7,8,9,10,11]. The availability of P for plants is governed by the degree of attachment to cations (Fe, Al, and Ca) and carbon moieties present in the soil, resulting in different levels of P availability for plant uptake [7,12]. In low-P soils, plants develop a myriad of chemical, biological, and biochemical mechanisms to increase P acquisition [13,14,15]. Rhizosphere acidification and the exudation of organic anions and phosphatase enzymes are deemed the most important processes taking place in the rhizosphere of P-deficient plants [16,17,18]. The exudation of protons from plant roots is used by P-deficient plants to acidify the soil and solubilize inorganic P [19,20,21,22]. Organic anions act via acidification, chelation, and exchange reactions to desorb sparingly available inorganic and organic P forms [21,23,24,25], while phosphatase enzymes released by plants and microorganisms contribute to the cleavage of organic P in order to supply available P to the soil solution [18,26,27,28]. Many of these mechanisms of P mobilization may also be duplicated by microbes [10,28,29,30]. Moreover, some authors have pointed out that organic anions and phosphatase enzymes can work together to mobilize organic P [31,32]. The model described by Clarholm et al. [33] highlighted this synergetic contribution in what is called the unbutton model.

Phosphorus dynamics are affected by plant species [19,34,35,36,37]. For instance, legume crops have been found to mobilize more stable P pools than cereals, reflecting divergent P acquisition strategies [38]. Ye et al. [37] found that P-efficient wild barley cultivars relied on acid phosphatase activity to mobilize different organic P fractions. Other authors highlighted the contribution of organic anions in the acquisition of sparingly available inorganic P forms in the rhizosphere of legumes such as chickpea, white lupin, and red clover [39,40,41,42]. However, the role of organic anions in enhancing P acquisition across plant species is unclear and has sometimes been found to be minor [43,44,45,46]. For instance, Pearse et al. [44] pointed out that organic anion exudation by different plant species was not consistently related to their ability to mobilize sparingly inorganic P forms, whereas Pandey et al. [47] and Ryan et al. [48] failed to find a strong correlation between plant yield, plant P content, and the release of organic anions. Additionally, most of the studies investigating organic anions and their impact on P availability have been carried out on sterilized sand or nutrient solutions, thus hampering our understanding of their role in real soil conditions [23,49,50]. Likewise, phosphatase activity was not always correlated with P deficiency [51,52,53], while alkaline phosphatase enzymes are deemed to be principally derived from soil microbes [18,54,55,56]. Therefore, a more accurate understanding of the mechanisms of P acquisition by legumes and grasses is a prerequisite for their use in intercropping systems or as potential green manures for the acquisition and recycling of P in soil–plant systems [14,30,57,58,59].

In a N-limited environment, the application of N fertilizers was found to increase microbial activity, together with an enhancement of phosphatase activity and rhizodeposition [60,61,62]. In a P-deficient soil, adding N shifts growth to P limitation, thereby activating mechanisms to make P available. It is widely reported that the addition of inorganic P to P-deficient soils inhibits phosphatase activity [18,63,64]. Nevertheless, the addition of N and P has given inconsistent results in terms of phosphatase activity and microbial biomass. For example, a meta-analysis on the effect of N addition on P limitation revealed an enhancement of phosphatase activity by 24%, while no effects on available soil P and microbial P were observed [65]. Conversely, Liu et al. [66] and Yang et al. [67] showed that P addition increased alkaline phosphatase activity, while N addition had an opposite effect.

Previous experiments combining the addition of N and P and investigating P dynamics have been focused on forest ecosystems due to increased concerns about atmospheric N depositions and their impact on soil P availability [35,62,67,68]. In contrast, studies on legume and grass species have been mostly carried out in inert sand or soils but with either N or P addition [37,40,69,70]. The application of N and P fertilizers is a common practice among farmers to increase pasture production, and is more representative of what happens in the field [71]. However, few studies have investigated the complicating factor of combined N and P addition on soil P dynamics. Here, we contrasted four plant species with a focus on two legumes and two grasses sown in small PVC tubes to accelerate P cycling and encourage easy detection of plants’ responses to P deficiency. Moreover, in this study we aimed to identify the most important mechanisms driving P acquisition in different plant species and potential changes due to nutrient addition in a low P pasture soil. We hypothesized that: (1) nutrient addition could impact rhizosphere properties, which in turn may affect soil P acquisition; (2) plant species could mobilize different soil P fractions, regardless of their chemical availability, using phosphatase enzymes or organic anions.

## 2. Results

### 2.1. Plant Biomass and Plant P Contents

Phosphorus addition had a significant impact on plant growth. In terms of the total plant biomass (root + shoot), plant species reacted to P addition as follows: white clover > ryegrass > wheat > blue lupin. In the P treatment, shoot biomass was increased by 413, 449, 118, and 146% for white clover, ryegrass, wheat, and blue lupin, respectively; whereas root biomass was increased by 388, 164, 27, and 10%, respectively, compared to the control (Table 1). In the NP treatment, shoot biomass was increased by 635, 504, 146, and 20% for white clover, ryegrass, wheat, and blue lupin, respectively. Root biomass followed the same trend and was increased by 510, 393, 78, and 19% for white clover, ryegrass, wheat, and blue lupin, respectively. On the other hand, N treatment showed no significant differences in plant biomass compared to the control, irrespective of plant species, whereas the NP treatment resulted in higher plant biomass compared to the other treatments, especially for grass species. Across treatments, the shoot-to-root ratio values were highest in blue lupin, showing values greater than 1, while the values for this ratio were 0.5, 0.7, and 0.6 for white clover, ryegrass, and wheat, respectively. Plant P concentrations were affected by plant species and nutrient addition. Blue lupin had the highest shoot P concentration (1.2 mg g^−1^), while white clover showed the greatest root P concentration, regardless of treatment (1.5 mg g^−1^). Across plant species, the responses of shoot and root P concentrations were similar and significantly increased under P and NP treatments, although lower P concentrations were observed under the NP treatment, while no changes were noted in the N treatment compared to the control (Table 1). Total P content was enhanced by 5.3-, 3.7-, 1.5-, and 0.4-fold after P addition in white clover, ryegrass, wheat, and blue lupin, respectively; whereas in the NP treatment this parameter increased by 6.3-, 4.3-, 1.4-, and 0.3-fold for the same plant species (Table 1). Additionally, plant P content was statistically similar under P and NP treatments on one hand, and for the control and N treatments on the other hand.

### 2.2. Rhizosphere pH

**Figure 1:**
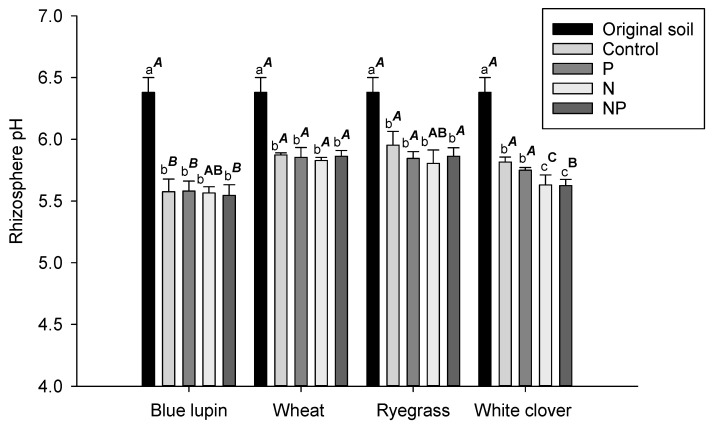
Rhizosphere pH values for blue lupin, wheat, ryegrass, and white clover for the control (0N, 0P), P (0N, 50P), N (300N, 0P), and NP (300N, 50P) treatments compared to the pH of the original soil. Different letters represent a significant difference (*p* < 0.05) among nutrient treatments for the same plant. Different superscript letters represent a significant difference (*p* < 0.05) among plant species for the same nutrient treatment.

Soil pH values measured in the rhizospheres of all plant species significantly decreased compared to the pH of the original soil (pH 6.4). A more pronounced decrease was observed in the rhizospheres of blue lupin and white clover, where the pH values dropped to averages of 5.6 and 5.7, respectively; followed by ryegrass and wheat, with pH values reaching an average of 5.9 (Figure 1).

### 2.3. Microbial Biomass P

**Figure 2:**
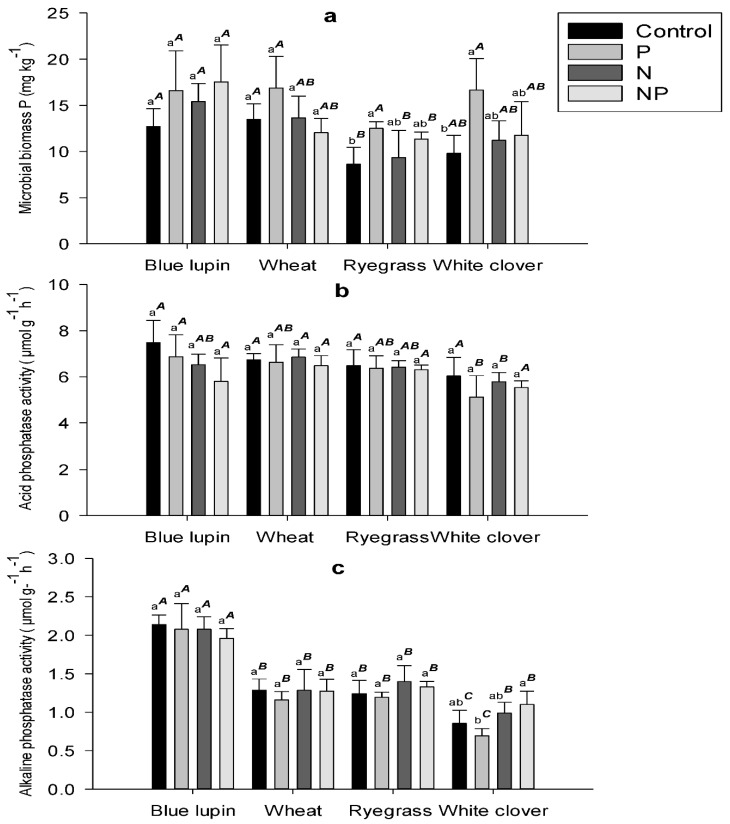
(**a**) Microbial biomass P, (**b**) acid phosphatase activity, and (**c**) alkaline phosphatase activity values in the rhizospheres of blue lupin, wheat, ryegrass, and white clover for the control (0N, 0P), P (0N, 33P), N (200N, 0P), and NP (200N, 33P) treatments. Different letters represent a significant difference (*p* < 0.05) among nutrient treatments for the same plant. Different superscript letters represent a significant difference (*p* < 0.05) among plant species for the same nutrient treatment.

Plant species had a significant effect on microbial biomass P, with ryegrass exhibiting the lowest concentration across treatments. P addition increased microbial biomass P across plant species, with significant increases of 45% and 70% for ryegrass and white clover, respectively (Figure 2a). N and NP treatments increased microbial biomass P across plant species but there were no significant differences between those treatments and the control.

### 2.4. Phosphatase Activity

Acid phosphatase activity was not affected by plant species or nutrient treatments, showing an average value of 6 µmol g^−1^ h^−1^ (Figure 2b). However, alkaline phosphatase activity was significantly impacted by plant species, but was similar across nutrient treatments (Figure 2c). In fact, alkaline phosphatase activity for blue lupin (average of 2 µmol g^−1^ h^−1^) was 60, 55, and 120% higher compared to wheat, ryegrass, and white clover, respectively. Acid phosphatase activity was on average 4.4-fold higher than alkaline phosphatase activity in this study.

### 2.5. Organic Anions

**Figure 3:**
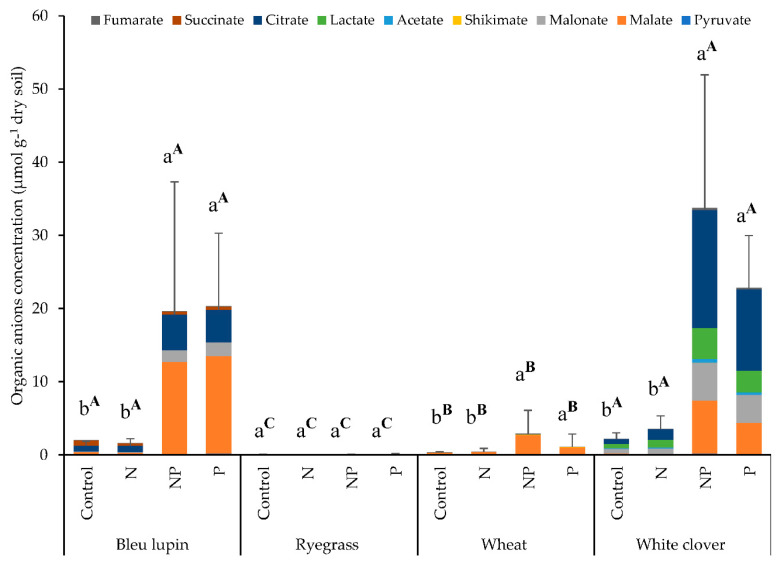
Concentrations of organic anions and their compositions in the rhizospheres of blue lupin, wheat, ryegrass, and white clover for the control (0N, 0P), P (0N, 33P), N (200N, 0P), and NP (200N, 33P) treatments. Different letters represent a significant difference (*p* < 0.05) among nutrient treatments for the same plant. Different superscript letters represent a significant difference (*p* < 0.05) among plant species for the same nutrient treatment.

Organic anions released in the rhizosphere varied significantly among plant species and nutrient treatments (Figure 3). A total of nine organic anions were detected in this study, including citrate, malate, malonate, acetate, pyruvate, lactate, succinate, fumarate, and shikimate (Figure 3). Legumes released significantly more organic anions than grasses. In fact, white clover had the highest concentration of organic anions, with an average of 16 µmol g^−1^ dry soil, followed by blue lupin (11 µmol g^−1^ dry soil), wheat (1.2 µmol g^−1^ dry soil), and ryegrass (0.05 µmol g^−1^ dry soil). Interestingly, after P addition (P and NP treatments), the concentrations of organic anions increased by 11-, 10-, 5-, and 2-fold in the rhizospheres of blue lupin, white clover, wheat, and ryegrass, respectively, compared with the control and N treatments (Figure 3). The same trend was observed when the concentration of organic anions was expressed by unit of root dry matter (Figure S1).

In the rhizosphere of white clover, citrate was the prominent organic anion released, representing 42% of total organic anion release, followed by malonate (21%), acetate (20%), and malate (13%); whereas the blue lupin rhizosphere was enriched with citrate (41%), followed by malate (36%) and malonate (7%). In the wheat rhizosphere malate accounted for 81% of the total organic anions, while pyruvate was the only organic anion reported in the rhizosphere of ryegrass due to the inconsistencies found for the other organic anions detected (Figure 3).

### 2.6. Rhizosphere P Fractions

**Figure 4:**
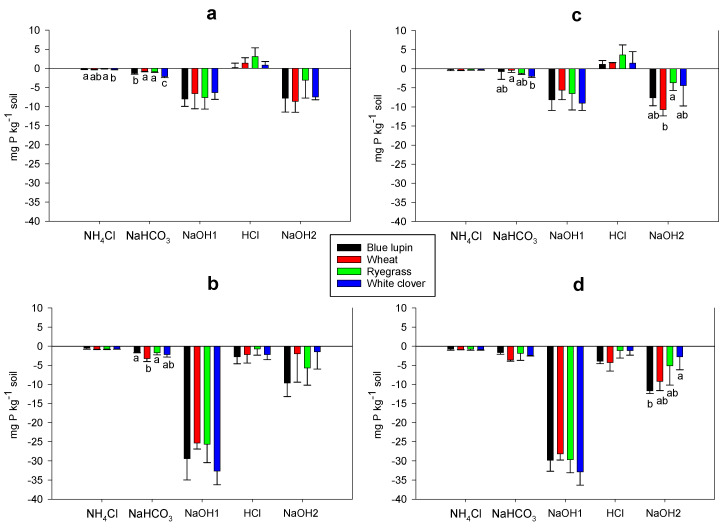
Changes (depletion or accumulation) in different inorganic P fractions with the control conditions (**a**), P treatment (**b**), N treatment (**c**), and NP treatment (**d**) in the rhizospheres of blue lupin, wheat, ryegrass, and white clover. Different letters denote a significant difference (*p* < 0.05) among plant species for a given nutrient treatment.

In the control and N treatments, the four plant species depleted all inorganic P fractions, except the Ca-P bound fraction (HCl-Pi), which showed an accumulation. Higher depletion rates of moderately labile Pi (NaOH1-Pi) and stable Pi (NaOH2-Pi) were observed across plant species, with average rates of 8 and 7 mg kg^−1^, respectively (Figure 4a,c). Interestingly, in P and NP treatments, plant species were able to deplete all inorganic P fractions, but with a more pronounced depletion of moderately labile Pi (NaOH1-Pi), which showed a 4-fold increase compared to under conditions of no P supply (Figure 4b,c).

**Figure 5:**
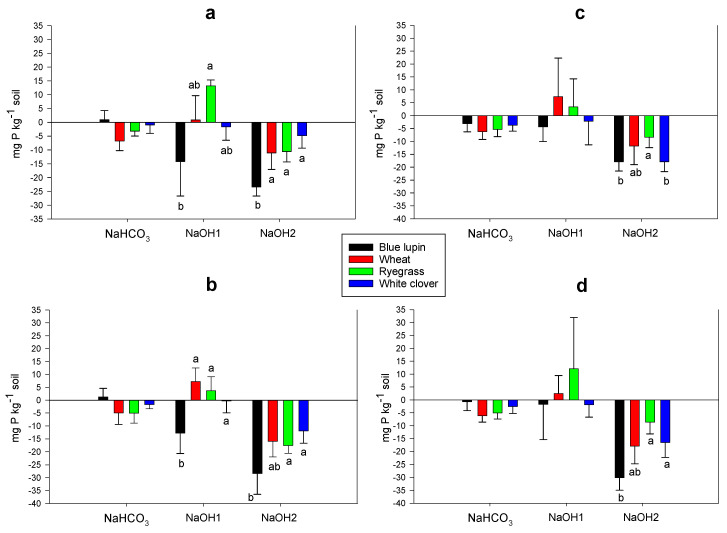
Changes (depletion or accumulation) in different organic P fractions with the control conditions (**a**), P treatment (**b**), N treatment (**c**), and NP treatment (**d**) in the rhizospheres of blue lupin, wheat, ryegrass and white clover. Different letters denote a significant difference (*p* < 0.05) among plant species for a given nutrient treatment.

The labile Po fraction (NaHCO_3_-Po) was depleted across plant species and nutrient treatments, except for blue lupin, which accumulated this fraction in the control and the P treatments. Blue lupin significantly depleted moderately labile Po (NaOH1-Po) (around 14 mg kg^−1^ soil) under the control and P treatments, while wheat and ryegrass accumulated this fraction regardless of the nutrient treatment (Figure 5a–d). Without P supply, blue lupin, white clover, wheat, and ryegrass depleted stable Po (NaOH2-Po) by an average of 21, 11, 11, and 9 mg kg^−1^ soil, respectively, whereas this depletion was increased by 1.5-fold across plant species after P addition. No changes were observed in the residual P in the rhizospheres of the four plant species compared to the original soils, thus they were not reported in the corresponding tables and figures. Moreover, N addition did not have any impact on P dynamics, and changes in P fractions were similar in comparison to the control treatment.

## 3. Discussion

### 3.1. Plant Responses to Nutrient Addition

Phosphorus addition increased plant biomass together with total P content regardless of the plant species. Moreover, white clover exhibited the highest increase in root and shoot biomass in response to P addition, while blue lupin was the least affected by P supply, but showed the highest shoot P concentration across plant species. The high P demand of white clover may explain the high response of this crop to P addition [59,72]. Having the highest shoot-to-root ratio, blue lupin demonstrated its aptitude for soil P acquisition, as confirmed by previous studies [73,74,75]. Nitrogen addition did not increase plant biomass nor improved total P content values across plant species. This suggests that N was not a limiting factor for plant growth in this study. In fact, legume plants did not show any root nodulation in our experiment. The combined addition of N and P (treatment NP) in this study increased plant biomass for grass plants compared to the other treatments, probably due to a synergetic effect of N and P on dry matter yield and an improvement of nitrogen uptake after alleviation of P deficiency in this treatment [76]. We acknowledge that the small tube volume used may have restricted plant growth in this study.

### 3.2. Rhizosphere pH

Changes in rhizosphere pH have been reported in P-deficient soils and in response to nutrient addition, with decreases and increases according to plant species, soil types, initial soil pH, and nutrient forms [77,78,79,80,81]. In this study, rhizosphere pH values were affected by plant species, but were similar across nutrient treatments. Among species, legumes are known to acidify the rhizosphere soil via the release of protons to compensate for excess cation uptake [78,82,83]. Indeed, our findings illustrated that legumes, especially blue lupin, exhibited lower rhizosphere pH values compared to the other plant species. On the other hand, the effect of P and N addition on rhizosphere pH was unclear in this study.

### 3.3. Microbial Biomass P

Phosphorus addition increased microbial biomass P across plant species and especially in ryegrass and white clover. This corroborates the results reported by Dodd et al. [84] after adding inorganic P fertilizers to a similar soil as that used in our study. They concluded that an increase in microbial biomass P was removing P from the soil solution. In fact, when sufficient carbon and N are present in the soil, microorganisms tend to immobilize available inorganic P in order to maintain the stoichiometric ratio of their biomass [85,86]. Microbial biomass P was not improved by N addition across plant species, which is in line with the findings reported by Deng et al. [65] in their meta-analysis. As discussed, this result emphasized that N was not a limiting factor in our soils and that P was the only limiting nutrient for both microbial and plant growth.

### 3.4. Phosphatases Activity

Acid phosphatase activity was not affected by plant species or nutrient addition in this study. Several reports have shown that P deficiency does not always result in an increase in phosphatase activity, while the addition of soluble P did not decrease phosphatase enzymes in different agroecosystems [51,52,53,67,69]. In acidic soils, the activity of acid phosphatase enzymes is considered to be higher compared to other phosphomonoesterases [27,87]. In our study, rhizosphere pH values were affected by plant species but were still acidic for the four plant species. This could explain why no differences were detected in acid phosphatase activity. Moreover, phosphatase enzymes can be adsorbed into organic matter and the soil solid phase—especially clay minerals [18,27]—complicating the interpretation of enzyme assays, as they could include the active, latent, and adsorbed enzymes [88].

In contrast to acid phosphatase activity, alkaline phosphatase activity was affected by plant species, with blue lupin exhibiting significantly higher activity in its rhizosphere. No obvious nutrient effects were observed across plant species for these enzymes. Alkaline phosphatase enzymes are believed to be mainly released by microorganisms [18] and to be affected by plant species, microbial community composition, and rhizodeposition [55,89,90]. For instance, Wasaki et al. [55] found that the rhizosphere of P-deficient white lupin plants exhibited an increase in the alkaline phosphatase activity derived from microbes, together with potential decreases in different organic P forms, such as phytates. Moreover, alkaline phosphatase enzymes are ubiquitous in soils, but their activity is somehow influenced by the quantity and quality of rhizodeposits [55,56,91,92]. Our results showed a strong depletion of stable organic P (NaOH2-Po) in blue lupin, coupled with a higher alkaline phosphatase activity. Although our data showed that blue lupin had the highest alkaline phosphatase activity, microbial biomass P values were not significantly different among the four plant species. These findings indicate that blue lupin may have shaped its microbial niche towards a cohort of microbes dedicated to the release of alkaline phosphatase enzymes, probably via rhizodeposition [55,92]. Nevertheless, more detailed assessments of microbial diversity and phosphatase genes abundance and expression are required to verify this hypothesis. Bering in mind that phosphatase assays represent only the potential activity of phosphatase enzymes, since they are performed in optimal pH, soil slurry, and temperature conditions [18,27,93], the interpretation of these results must be done cautiously.

### 3.5. Organic Anions

Organic anion release is a physiological adaptation of plants to P deficiency [94], the contribution of which to enhancing P availability has been widely demonstrated [21,39,95]. Our results showed the presence of 9 organic anions in the rhizosphere of the four plants studied, with citrate, malate, and malonate being the most important organic anions released. Legumes released higher concentrations of organic anions in their rhizospheres compared to grass plants, corroborating previous studies reporting that legumes relied on organic anions to increase their P acquisition [39,40,96,97]. Citrate, malate, and malonate have been described to be efficient in the desorption, chelation, and complexation of Fe and Al oxides to liberate P for plant uptake [23,24,25,42]. Most of the inorganic P added in our study was found in the NaOH-P fraction, suggesting that P was bound to Fe and Al oxides (Table S1). Therefore, the depletion of sparingly labile inorganic P (NaOH-Pi) observed in the rhizospheres of the four plants could be attributed to the organic anion release. However, our results showed contrasting amounts of organic anions measured in the rhizospheres of the four plant species, while no significant differences were observed in the depletion of NaOH1-Pi and NaOH2-Pi fractions among plant species. These findings suggest that organic anions were not related to P acquisition of sparingly labile inorganic P (NaOH-Pi) in our study and that some other parameters might have been involved in this acquisition, such as rhizosphere pH and root morphology. Indeed, several scholars have highlighted the fact that organic anions play a minor role in inorganic P acquisition and that plant species exhibit considerable variations in the release of organic anions, even within the same genus [23,44,97]. For instance, Pearse et al. [44] found that there was no consistent relationship between the ability to mobilize different inorganic P forms and carboxylate exudation from plant roots, whereas Wang et al. [45] pointed out that organic anion release for wheat, oat, potato, and canola plants did not improve P availability and was not related to shoot P uptake.

The expression of organic anions by unit of root dry matter has given similar results as by soil dry matter, emphasizing that organic anions released after P addition were not solely derived from plants, but could have come from other sources (Figure S1). Most of the studies looking at organic anion release and its influence on P availability and plant P uptake have been carried out on sand or liquid cultures. Therefore, the impact of soil organic matter and soil microorganisms on organic anion release is still not well understood [23,45,50]. It has been found that inorganic P inputs in grassland soils increased microbial activity, thus triggering soil organic matter degradation and carbon mineralization [98,99,100]. In fact, recent findings have showed that the addition of inorganic P increased microbial respiration and the desorption of organic carbon [101]. Thus, the increase in organic anions found after P addition in our study could be ascribed to an increased priming effect on soil organic matter by microorganisms. Up to 50% of soil microbes are able to mobilize soil P by duplicating different mechanisms used by plants, such as the exudation of organic anions and the release of phosphatase enzymes [11,23,29,30,102]. Plants and microorganisms are in competition for available nutrients in the soil solution, especially in low fertility conditions [26,103,104]. Our results showed that the addition of inorganic P increased microbial biomass P, indicating an immobilization of inorganic P by microbes. Thus, plants and microorganisms may have increased their organic anion release to mobilize the additional P needed to meet their demands [105]. Nevertheless, the relative contribution of microbes and plants to this mechanism needs further research [29].

In summary, our results suggest that (1) organic anions played a minor role in the acquisition of sparingly labile inorganic P, and that (2) organic anions measured in the rhizosphere of the four plant species after P addition could be partly coming from soils microbes or might be derived from the microbial priming effect on soil organic matter.

### 3.6. Rhizosphere P Fractions and P Acquisition

Inorganic P can be adsorbed into the active mineral surfaces of Fe, Al, and Ca, thus decreasing its availability for plant uptake [4,5,6]. Our results showed that the four plant species depleted different inorganic P fractions, including labile (NH_4_Cl-Pi and NaHCO_3_-Pi), moderately labile (NaOH1-Pi), and stable inorganic P (NaOH2-Pi). In contrast, after P addition, a 4-fold increase in the depletion of moderately labile inorganic P (NaOH1-Pi) was observed, most likely due to an increased plant P uptake and microbial immobilization. Depletion of labile Pi fractions has been reported by different authors [37,69,74,106], while Chen et al. [107] found that the NaOH-Pi fraction was accessible by plants and able to supply available P in a range of temperate grassland soils. In the literature, the depletion of NaOH-Pi was mainly related to the action of organic anions [21,23,42]. In our study, the depletion of this fraction was similar across plant species, although different amounts of organic anions were found in the rhizosphere of the four plant species. As discussed previously, this indicates that the depletion of this fraction was not directly related to organic anions, and that some other attributes such as root traits and rhizosphere pH may have been involved in the depletion of this fraction. Our results showed that calcium-P (HCl-Pi) increased in the absence of P input, which could be ascribed to a complexation of some inorganic P by free calcium cations present in the soil due to liming, while the depletion of this fraction after P addition may reflect an increased plant P uptake. Overall, our findings indicated that under P-deficient conditions and restricted soil volumes (rhizosphere soil), plants are able to mobilize both labile and recalcitrant inorganic P fractions to meet their P demands, regardless of the chemical availability of these fractions.

Organic P can represent a high proportion of total P in soils [108], especially in pastures where organic residues have little turnover and accumulate [8,9,109]. In our study, organic P represented more than 50% of the total P, which was mainly comprised of NaOH-Po, thus being a potential source of P for plant uptake [9,56,110,111]. Phosphatase enzymes are released by plants and microorganisms to mobilize organic P via the cleavage of carbon-P bounds, which release available orthophosphates into the soil solution [7,18]. From all the organic P fractions, stable organic P (NaOH2-Po) was the most depleted by the four plant species, although a 1.5-fold depletion was noted after P addition for this fraction, most likely due to increased plant uptake. Although listed as stable, this phenomenon is defined by resistance to chemical extraction and clearly not to action by plants or microbes. Therefore, this challenges the assumption that NaOH2-Po represents a recalcitrant P fraction and shows that this fraction could be biologically mobilized by plants in the rhizosphere. More importantly, our findings showed that blue lupin significantly depleted this fraction compared to other plant species, which concurred with higher phosphatase activity in the rhizosphere of this plant. White lupin has been found to deplete organic P pools due to higher alkaline phosphatase activity being derived from microbes, probably due to the release of an array of carboxylases shaping its microbial community composition [55]. In another study carried out by Wei et al. [56], alkaline phosphatase enzymes released by a specific bacterial community in the rhizosphere of rice was linked to the depletion of organic P. Thus, we suggest that alkaline phosphatase enzymes may have been responsible for the higher aptitude of blue lupin to deplete stable organic P in this study. Phosphatase assays measure both the active and stabilized extracellular enzyme activity and represent the upper limit of phosphatase activity in soils [27,102]. Thus, we cannot directly link the higher alkaline phosphatase activity in the rhizosphere of blue lupin with the depletion of stable organic P. Further investigations are required using molecular and genomic tools to tease apart the contributions of these different sources of extracellular enzyme activity, as well as to decipher the role of alkaline phosphatase activity in organic P mobilization, especially in acidic soils. Labile organic P accumulated in blue lupin, while moderately labile organic P accumulated in grasses, probably due to an immobilization of some inorganic P by soil microbes [112], however we have no data to confirm this. Residual P levels changed little during our experiment, presumably due to its high recalcitrance together with the mobilization of other P pools [37,80].

Several studies have shown that in N-limited environments, N addition in the form of fertilizers or via atmospheric deposition can contribute to the depletion of different P fractions and can exacerbate P limitation due to increased phosphatase activity and rhizodeposition [35,60,61,62]. However, our study showed no effect of N addition on P dynamics or transformation. This is probably because N was not a limiting factor in our soil, and therefore it did not influence plant growth and rhizosphere processes related to P acquisition for the four plant species used in this experiment.

In our study, rhizosphere P fractions were compared to the soil at day 0; therefore, we acknowledge that some P depletions, especially in the labile P fractions, could be partly due to wetting and drying processes, although depletions across plant species were consistent. Moreover, we estimated the P supplied by seeds to be negligible compared to the P present in the soil (soil total P: 1236 mg kg^−1^) and probably did not impact soil P dynamics [113,114]. In fact, taking into consideration the number of seeds per pot and total P per seed, the quantities of P supplied by seeds were approximately 1, 0.6, 0.16, and 0.07 mg P pot^−1^ for blue lupin, wheat, ryegrass, and white clover, respectively.

## 4. Materials and Methods

### 4.1. Soil Preparation and Characteristics

The soil used in the experiment was a silt loam Waikiwi soil (USDA soil taxonomy: Inceptisol) from the Woodlands Research Station, 19 km east of Invercargill, New Zealand. Soil samples were collected from the 0–20 cm horizon of a permanent pasture that had not received P fertilizers for the last 25 years, air-dried and passed through a 2 mm sieve to remove any roots and plant materials. The soil had an Olsen P concentration of 4 mg kg^−1^ and an initial pH in water of 5.4 (1:2.5). Before starting the experiment, the soil was limed with CaCO_3_ at a rate of 4 tons per hectare in order to reach an optimal pH for plant growth (pH 6.4), then incubated at 25 °C for two weeks at 75% of field capacity. The soil received a basal fertilization consisting of 265 mg kg^−1^ of K (K_2_SO_4_), 30 mg kg^−1^ of Mg (MgO), 3 mg kg^−1^ Mn (MnCl_2_,4H_2_O), 2 mg kg^−1^ Zn (ZnCl_2_), 2 mg kg^−1^ Cu (CuCl_2_,2H_2_O), 3 mg kg^−1^ B (H_3_BO_3_), and 0.2 mg kg^−1^ Mo (Na_2_MoO_4_,2H_2_O). The other basic properties of the soil used in this study are summarized in the Table 2.

### 4.2. Experimental Design

For the purpose of this study, PVC tubes (48 mm internal diameter) were cut at 70 mm height, sealed from the bottom with a 20 µm nylon mesh, and filled with approximately 100 g of oven-dry soil to simulate a bulk density of 0.89 g cm^−3^. Before sowing, the soil received two levels of P-0 and 33 mg kg^−1^ as NaH_2_PO_4_ and two levels of N-0 and 200 mg kg^−1^ as urea, in a full factorial design resulting in the following four treatments: control (0 P, 0 N), P (33 P, 0 N), N (0 P, 200 N), NP (33 P, 200 N). The fertilizers were mixed thoroughly with the soil. These rates of N and P inputs reflected some common practices in fertilization programs in New Zealand pasture systems, especially dairy farms in the Canterbury region [71]. Four plant species, namely blue lupin (*Lupinus angustifolius*), white clover (*Trifolium repens* L., cv. Demand), perennial ryegrass (*Lolium perenne* L., cv. Samson), and wheat (*Triticum aestivum* L., cv. Graham), were used in this study. Twenty seeds of white clover and ryegrass and 6 of wheat and blue lupin were sterilized for 20 min in 2% sodium hypochlorite, washed thoroughly with deionized water, pre-germinated in the dark in wet filer papers, and then sown in the tubes. After emergence, seedlings were thinned to 15 plants for white clover and ryegrass, 5 for wheat, and 4 for blue lupin. The purpose of using a high seed density and a small volume of soil was meant to maximize soil exploitation by roots (all the soil in the tube was considered as rhizosphere soil), accelerate P cycling, and encourage easy detection of plant species responses to P deficiency. A pre-experiment was carried out to determine the optimal number of seeds for each plant species. White clover and blue lupin (legumes) were not inoculated in this study because of the presence of their respective rhizobia strains in New Zealand pasture soils. The tubes were placed in a randomized bloc design in a glasshouse between August and October 2018 within a temperature range of 10 to 26 °C and a 16:8 dark/light ratio. There were four replicates of each treatment. The tubes were irrigated by capillarity via a sand bed covered with nylon mesh and connected to a water reservoir via a syphon system. The difference between the water level in the reservoir and the bottom of the tubes was kept at 5 cm height to supply enough water to the tubes during the experiment.

### 4.3. Plant and Soil Analyses

After 60 days of growth, plants were removed from the tubes and the rhizosphere soil was sampled. Due to the high root density, all the soil in the tube was considered as rhizosphere soil. The soil samples were then sieved through a 2 mm mesh sieve and divided into two portions. The first portion was air-dried for chemical analyses. The second was stored fresh at 4 °C for microbial biomass P and phosphatase enzyme measurements. Roots and any remaining soil were transferred to a plastic beaker, where they were gently soaked for two minutes in a known volume of 0.2 mM CaCl_2_ solution (50 and 100 mL for small and big root biomass samples, respectively), liberating a rhizosphere soil extract that was used for organic anion determination by HPLC [44,115]. Shoot and root materials were separated, rinsed with deionized water, oven-dried at 65 °C for 48 h, and then weighed. Plant material was ground to pass through a 1 mm sieve. Shoot and root P concentrations were determined by inductively coupled plasma atomic emission spectroscopy (ICP-EOS) after digestion with a mixture of HNO_3_ and H_2_O_2_ [116]. Phosphorus contents in shoots and roots were calculated by multiplying P concentrations by the dry matter of the corresponding plant part.

Rhizosphere pH values were measured after shaking 4 g of air-dried soil with deionized water for 1 h in a 1: 2.5 soil to solution ratio. Microbial biomass P was determined following the fumigation-extraction method described by Brookes et al. [117], along with the recommendations of Morel et al. [118]. In short, 1 g of field moist soil was weighed in triplicate (sets A, B, and C). Set A was fumigated using CHCl_3_ for 24 h and extracted by 20 mL of 0.5 M NaHCO_3_ for 30 min. Set B was non-fumigated, kept for 24 h in the same conditions, and extracted with 20 mL of 0.5 M NaHCO_3_ for 30 min. Set C was also non-fumigated and kept for 24 h in the same conditions, but extracted with 20 mL of 0.5 M NaHCO_3_ spiked with 25 mg kg^−1^ P for 30 min. All the extracts were then centrifugated at 3300 rpm for 10 min and filtered through Whatman number 2 for colorimetry measurements of inorganic P following the molybdate-ascorbic acid method [119]. Microbial biomass P was calculated according to the following equation:

Microbial Biomass P (mg kg^−1^) = 25 × (P_A_ − P_B_)/(0.40 × (P_C_ − P_B_)) (1)

Acid and alkaline phosphomonoesterase activity were determined according to the procedure described by Tabatabai [27]. The method consists of adding 4 mL of modified universal buffer (MUB; pH 6.5 for acid phosphatase and pH 11 for alkaline phosphatase) to 1 g of moist soil, followed by adding 1 mL of substrate p-nitro phenyl phosphate (50 mM) and then incubating the mixture for 1 h at 37 °C (the final concentration of p-nitro phenyl phosphate (substrate) was 10 mM based on the total volume used (5 mL)) [93]. The reaction was halted by the addition of 4 mL 0.5 M NaOH and 1 mL of 0.5 M CaCl_2_. The extract was then centrifugated at 3300 rpm for 15 min, filtered, and diluted as needed for spectrophotometry readings at 410 nm. The activity of acid and alkaline phosphatases enzymes was considered as potential or apparent [93,102]. For organic anion analysis, a subsample of the rhizosphere soil extract was filtered through a 0.45 µm Phenex regenerated cellulose syringe (Phenomenex, Torrance, CA, USA), then one drop of diluted (1:100) Micropur (10 mg/L, Katadyn products, Lindau, Switzerland) was added to inhibit microbial decomposition [45,120,121]. One drop of concentrated orthophosphoric acid was added to each sample, which were then stored at −20 °C until analysis by HPLC. The remaining rhizosphere extract was then filtered through a Whatman number 1 filter paper and oven-dried at 70 °C for three days to determine the rhizosphere soil dry weight [45,122]. Organic anions were determined using a Prevail^TM^ organic acid column (300 × 4.6 mm, 5 µm particle size, Grace Davison Discovery Sciences) with 25 mM KH_2_PO_4_ (pH: 2,35) as a mobile phase, 0.6 mL min^−1^ as the flow rate at 50 °C, with an injection volume of 30 µl and a detection wavelength of 210 nm. Different organic anions were identified by the comparison of the retention times and absorbance rates of known organic anions standards.

Soil P fractionation is an extraction method used to separate chemically bound P fractions, which informs on the extent to which these fractions are available for plants [4,110,123,124]. In our study, soil P fractionation was conducted on the soil at day 0 and on the rhizosphere soil after plant growth. For this purpose, the procedure developed by Hedley et al. [4] was used, along with the modifications from Condron et al. [111] and Condron and Newman [123]. Briefly, 0.5 g of air-dried soil was sequentially extracted using 10 mL of 1 M NH_4_Cl, 0.5 M NaHCO_3_ (pH 8,5), 0.1 M NaOH, 1 M HCl, and a second 0.1 M NaOH (to extract P in the micro-aggregates and the P protected by calcium) [123], then shaken for 16 h each time in an end-over shaker to extract the following fractions: NH_4_Cl-P, NaHCO_3_-P, NaOH1-P, HCl-P, and NaOH2-P. The last residue was dried at 50° C and digested with concentrated H_2_SO_4_ and H_2_O_2_ to determine residual P values [125]. Inorganic P (Pi) in the alkali extracts (NaHCO_3_-Pi, NaOH1-Pi, NaOH2-Pi) was determined according to Dick and Tabatabai [126] and He and Honeycut [127] to avoid overestimation of inorganic P due to mineralization of labile organic P. The method of Murphy and Riley [119] was used to determine Pi in the acid extracts (NH_4_Cl-Pi, HCl-Pi, and residual Pi). Total P (Pt) in the alkali extracts was measured using ICP-EOS according to do Nascimento et al. [128], while organic P (Po) was calculated as the difference between Pt and Pi in each fraction. The total soil P was calculated as the sum of all nine fractions. The availability of a fraction for plant uptake was presented based on its depletion or accumulation from the rhizosphere [34], which was calculated as the difference between P in the soil at day 0 and in the rhizosphere soil after plant growth for each treatment (Figure 4 and Figure 5). The distributions of different P fractions in the four treatments at day 0 are reported in Table S1.

### 4.4. Data Analysis

Data were subjected to a two-way analysis of variance to determine the effects of N and P addition (nutrient effect) and plant species (plant effect) on plant and soil parameters. To test the effects of plant species on the depletion of different rhizosphere P fractions, we used one-way analysis of variance or the Kruskall-Wallis test when homoscedasticity was not met. The Tukey test was used to distinguish significant differences between treatment means at 5% probability. In cases of variance heterogeneity, the Games-Howell post hoc test was used to separate homogenous groups at 5% probability. All statistical analyses were performed with SPSS 25 (IBM Corp, Armonk, NY, USA). Different organic anions were detected in the rhizosphere of ryegrass, but due the very high variability between the replicates for some organic anions, only pyruvate was reported for this species; all other organic anions were omitted from the calculation of the total organic anion concentrations.

## 5. Conclusions

Plant species and nutrient availability are among key parameters governing soil P acquisition. Our study showed that legumes and grasses reacted differently to P addition in terms of plant biomass, total P content, and rhizosphere processes involved in soil P acquisition. Nitrogen addition did not affect any parameters measured in this study, whereas P addition increased microbial biomass P and organic anion release across plant species, but did not affect acid phosphatase activity and rhizosphere pH. Alkaline phosphatase activity was higher under blue lupin. Our results showed that in restricted soil volumes such as the rhizosphere, plants can mobilize different inorganic and organic P fractions, regardless of their potential chemical availability, as assessed by sequential P fractionation. Moderately labile inorganic P (NaOH1-Pi) and stable organic P (NaOH2-Po) were the most depleted fractions across plant species, with blue lupin exhibiting higher ability to mobilize the later P pool. Although releasing different amounts of organic anions, the four plant species showed similar depletion rates of inorganic P fractions in their rhizospheres. We conclude that organic anions played a minor role in inorganic P acquisition for the four plant species investigated in this study, whereas the strong depletion of stable organic P observed in the rhizosphere of blue lupin was associated with alkaline phosphatase activity.

## Figures and Tables

**Table 1:** Root and shoot biomass (g tube^−1^), root and shoot P concentration (mg g^−1^), and total P content (mg tube^−1^) values for blue lupin, wheat, ryegrass, and white clover under the different nutrient treatments.

	Root Biomass	Shoot Biomass	Root P	Shoot P	Plant P Content
**Blue Lupin**					
Control	1.2 ± 0.1 b ^1^	2.2 ± 0.1 b	1.0 ± 0.0 b	1.1 ± 0.0 c	3.54 ± 0.25 c
P	1.7 ± 0.2 a	2.4 ± 0.1 a	1.1 ± 0.1 a	1.3 ± 0.1 b	4.84 ± 0.69 a
N	1.4 ± 0.3 b	2.1 ± 0.1 b	0.9 ± 0.0 c	1.0 ± 0.1 d	3.26 ± 0.49 c
NP	1.5 ± 0.2 a	2.6 ± 0.2 a	1.0 ± 0.1 b	1.4 ± 0.1 a	4.67 ± 0.70 b
**Wheat**					
Control	0.9 ± 0.0 c	1.1 ± 0.0 c	0.8 ± 0.1 b	0.4 ± 0.0 c	1.25 ± 0.08 c
P	1.9 ± 0.1 b	1.4 ± 0.0 b	1.1 ± 0.0 a	0.8 ± 0.0 a	3.21 ± 0.09 a
N	0.9 ± 0.1 c	1.1 ± 0.1 c	0.8 ± 0.1 b	0.4 ± 0.0 c	1.28 ± 0.13 c
NP	2.2 ± 0.2 a	2.0 ± 0.2 a	0.9 ± 0.1 b	0.6 ± 0.0 b	3.08 ± 0.24 b
**Ryegrass**					
Control	0.4 ± 0.0 c	0.2 ± 0.0 c	1.2 ± 0.0 b	0.7 ± 0.0 b	0.58 ± 0.04 c
P	1.0 ± 0.1 b	0.8 ± 0.1 b	1.3 ± 0.0 a	1.1 ± 0.0 a	1.65 ± 0.13 b
N	0.4 ± 0.2 c	0.3 ± 0.1 c	1.2 ± 0.1 b	0.7 ± 0.0 b	0.73 ± 0.27 c
NP	1.9 ± 0.2 a	1.1 ± 0.0 a	1.0 ± 0.1 c	0.6 ± 0.0 c	2.52 ± 0.25 a
**White clover**					
Control	0.2 ± 0.0 ^1^ c	0.2 ± 0.0 b	1.6 ± 0.1 a	0.7 ± 0.0 b	0.38 ± 0.03 b
P	1.0 ± 0.1 b	0.7 ± 0.1 a	1.5 ± 0.0 b	0.9 ± 0.0 a	2.03 ± 0.17 a
N	0.3 ± 0.0 c	0.2 ± 0.0 b	1.5 ± 0.0 b	0.7 ± 0.0 b	0.46 ± 0.08 b
NP	1.5 ± 0.1 a	0.9 ± 0.0 a	1.3 ± 0.0 c	0.8 ± 0.1 a	2.38 ± 0.39 a

^1^ Values represent the mean of four replicates ± standard errors. Different letters in columns denote a significant difference (*p* < 0.05) among nutrient treatments for a given plant.

**Table 2:** Basic physiochemical properties of the Waikiwi soil used in the study.

Soil pH	Total C	Total N	Total P	Olsen P	Exchangeable Potassium	Exchangeable Al	Soil Texture (%)		
	g C kg^−1^ soil	g N kg^−1^ soil	mg P kg^−1^ soil	mg P kg^−1^ soil	me/100 g soil	mg kg^−1^ soil	Sand	Silt	Clay
5.4	44	3.9	1236	4	0.16	7.2	12.6	37.8	49.6

## Supplementary Materials

The following are available online at https://www.mdpi.com/2223-7747/9/9/1185/s1: Table S1: Distribution of different soil P fractions for the four treatments at day 0. The control and N treatments are reported in the same column as the P and NP treatments. Figure S1: Concentrations of organic anions expressed by unit of root dry matter in the rhizosphere of blue lupin, wheat, ryegrass, and white clover for the control (0N, 0P), P (0N, 33P), N (200N, 0P), and NP (200N, 33P) treatments. Different letters represent a significant difference (*p* < 0.05) among nutrient treatments for the same plant. Different superscript letters represent a significant difference (*p* < 0.05) among plant species for the same nutrient treatment.
