# Supplementary material for: Role of Organic Anions and Phosphatase Enzymes in Phosphorus Acquisition in the Rhizospheres of Legumes and Grasses Grown in a Low Phosphorus Pasture Soil
Source: Plants (Basel). 2020 Sep 11;9(9):1185. doi: 10.3390/plants9091185 (PMC7570192; doi:10.3390/plants9091185)
Supplement: Supplementary file 1 [file plants-09-01185-s001.pdf]

Table S1. Distribution of different soil P fractions in the four treatments at day 0. The control and N treatments were reported in the same column as was for the P and NP treatments.

	P concentration (mg kg^{-1})	
	**Control and N treatments**	**P and NP treatments**
NH_4Cl-Pi	0.6 ± 0.1[1]	1.3 ± 0.2
$NaHCO_3$-Pi	7.5 ± 0.2	12.1 ± 0.2
$NaHCO_3$-Porg	56.5 ± 1.6	57.5 ± 3.4
NaOH1-Pi	103.3 ± 2.5	144.1 ± 5.5
NaOH1-Porg	455.9 ± 3.8	460.7 ± 7.9
HCl-Pi	9.5 ± 1.8	12.9 ± 1.8
NaOH2-Pi	59.3 ± 5.1	59.6 ± 3.5
NaOH2-Porg	146.5 ± 4.8	155.8 ± 8.0
Residual-P	396.7 ± 41.6	400.0 ± 35.9

[1]Values represent the mean of eight replicates ± SE

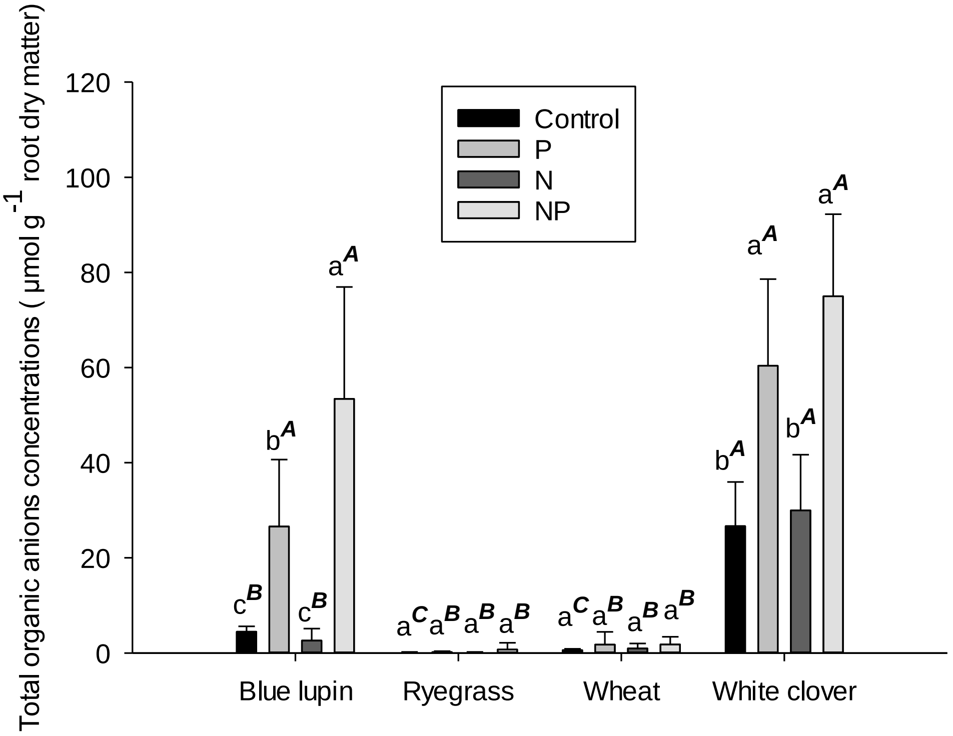

Figure S1. Concentration of organic anions expressed by unit of root dry matter in the rhizosphere of blue lupin, wheat, ryegrass and white clover for the control (0N, 0P), P (0N, 33P), N (200N, 0P), and NP (200N, 33P) treatments. Different letters represent a significant difference (*P*<0.05) among nutrient treatments for the same plant. Different superscript letters represent a significant difference (*P* <0.05) among plant species for the same nutrient treatment.
